# The antioxidative stress regulator Nrf2 potentiates radioresistance of oral squamous cell carcinoma accompanied with metabolic modulation

**DOI:** 10.1038/s41374-022-00776-w

**Published:** 2022-04-12

**Authors:** Yuichiro Matsuoka, Ryoji Yoshida, Kenta Kawahara, Junki Sakata, Hidetaka Arita, Hikaru Nkashima, Nozomu Takahashi, Masatoshi Hirayama, Masashi Nagata, Akiyuki Hirosue, Yoshikazu Kuwahara, Manabu Fukumoto, Ryo Toya, Ryuji Murakami, Hideki Nakayama

**Affiliations:** 1 Department of Oral and Maxillofacial Surgery, Faculty of Life Sciences, Kumamoto University, Kumamoto, 860-8556 Japan; 2 Division of Radiation Biology, Faculty of Medicine, Tohoku Medical and Pharmaceutical University, Miyagi, 981-8558 Japan; 3 Department of Molecular Pathology, Tokyo Medical University, Tokyo, 160-8402 Japan; 4 Department of Radiation Oncology, Faculty of Life Sciences, Kumamoto University, Kumamoto, 860-8556 Japan; 5 Department of Medical Radiation Sciences, Faculty of Life Sciences, Kumamoto University, Kumamoto, 862-0976 Japan

**Keywords:** Cancer metabolism, Oral cancer, Radiotherapy

## Abstract

Nuclear factor erythroid 2-related factor 2 (Nrf2), which regulates the expression of critical antioxidant proteins, was recently demonstrated to play a key role in cancer progression. Resistance to radiotherapy is a major obstacle in treating oral squamous cell carcinoma (OSCC). However, little is known about the association between Nrf2 and radioresistance in OSCC. Two OSCC cell lines (SAS and HSC-2) and their clinically relevant radioresistant (CRR) clones (SAS-R, HSC-2-R) were used. The effects of Nrf2 downregulation on radiosensitivity and the involvement of glycolysis in Nrf2-mediated radioresistance were evaluated. Immunohistochemistry of phosphorylated Nrf2 (p-Nrf2) was performed in 110 patients with OSCC who underwent preoperative chemoradiotherapy and surgery. Nrf2 was stably upregulated in CRR cells in vitro and in a mouse xenograft model. Moreover, elevated Nrf2 expression was associated with radioresistance. The enhancement of Nrf2-dependent glycolysis and glutathione synthesis was involved in the development of radioresistance. Additionally, p-Nrf2 expression was closely related to the pathological response to chemoradiotherapy, and its expression was predictive of prognosis in patients with advanced OSCC. Our results suggest that Nrf2 plays an important role in the radioresistance of OSCC accompanied by metabolic reprogramming. Targeting Nrf2 antioxidant pathway may represent a promising treatment strategy for highly malignant OSCC.

## Introduction

Radiotherapy is an important treatment modality for head and neck cancers, including oral squamous cell carcinoma (OSCC), which effectively ablates cancer cells by causing DNA damage through the generation of reactive oxygen species (ROS)^1^. However, treatment is often confounded by tumor resistance to radiotherapy, similarly as observed for surgical resection and chemotherapy^2^. Generally, cancer cells with antioxidant activity can escape the damaging effects of radiation by scavenging ROS, thereby leading to radioresistance^3^. Therefore, to improve the efficacy of radiotherapy, clarification of the characteristics of radioresistant OSCC cells is an important goal in radiation biology. Additionally, to improve patient outcomes, it is extremely important to explore novel biomarkers for guiding therapeutic management and predicting prognosis of OSCC. Moreover, the development of new treatment strategies is crucial.

The nuclear factor erythroid 2-related factor 2 (Nrf2)–Kelch-like ECH-associated protein 1 (Keap1) signaling pathway is one of the main cellular defense mechanisms against oxidative stress^4^. Under basal conditions, Nrf2 is sequestered in the cytoplasm by its repressor Keap1 and constitutively degraded through the ubiquitin–proteasome pathway^4^. Following cellular exposure to oxidative stress, Nrf2 escapes Keap1-mediated repression and translocates to the nucleus, in which it induces the transcription of numerous cytoprotective genes^4^. Recent studies revealed that activated Nrf2 is a key transcription factor associated with tumor development and progression in various cancers, including head and neck cancer^5–8^. Moreover, the Nrf2-mediated-antioxidant pathway is implicated in the chemoresistance and radioresistance of several malignancies, including OSCC^1,9^. Although we previously reported that Nrf2-mediated-antioxidant pathways play an important role in radiosensitivity in OSCC^10^, the detailed mechanisms have not been fully elucidated.

Since the discovery of the aerobic glycolysis system called the Warburg effect, the favorable and characteristic metabolic mechanisms for cancer cells have been reported^11–13^. Cancer cells activating metabolism accumulate abundant nutrients, including glucose and glutamine, and shunt their metabolites into anabolic pathways^11^. Mitsuishi et al. reported that stable Nrf2 activation promoted the reprogramming of metabolic activities, resulting in malignant phenotypes, such as cell proliferation in addition to enhancing cell protection^14^. Suppression of glycolysis metabolism by 2-deoxyglucose (2-DG), which inhibits glucose uptake, causes apoptosis-induced death in radioresistant cells^15–17^. Collectively, it is speculated we estimate that the high expression and nuclear accumulation of Nrf2 are directly involved in treatment resistance via Nrf2-dependent metabolic reprogramming.

In this study, we investigated the biological roles of Nrf2 in radiosensitivity in OSCC via in vitro and in vivo analyses.

## Materials and methods

### Parental cell lines and the establishment of clinically relevant radioresistant (CRR) cell lines

The human OSCC cell lines SAS and HSC-2 were obtained from the Japanese Collection of Research Bioresources bank of the National Institutes of Biomedical Innovation, Health and Nutrition (Osaka, Japan). The CRR cell lines SAS-R and HSC-2-R were established from SAS and HSC-2 cells, respectively, via gradual exposure to increasing X-ray doses of 0.5–2 Gy/day, as previously described^18^. These cell lines were cultured with DMEM supplemented with 10% FBS and maintained under humidified a 5% CO_2_ atmosphere at 37 °C.

### Animals and the in vivo experimental protocol

BALB/c-nu/nu female mice (6 weeks old) were purchased from Charles River Japan (Yokohama, Japan) and maintained at the Center for Animal Resources and Development of Kumamoto University, and animals were handled in accordance with the animal care policy of Kumamoto University. OSCC and CRR cells were trypsinized, washed with serum-free medium, resuspended in PBS, and adjusted to a density of 1 × 10^7^ cells/200 μl in PBS. Then, the cell suspensions were subcutaneously injected into the backs of nude mice (*n* = 4/cell line). When tumor volumes approached 150 mm^3^, experiments were started (day 0). Mice were exposed to a single X-ray dose of 10 Gy. Excluding the tumor regions, the bodies of the mice were protected from radiation by a lead shield. Seven days after irradiation, tumor tissues were excised, placed in sterile tubes, and immediately fixed in 10% formalin.

### Clinical samples from patients

For the histopathological analysis, primary OSCC tissue samples were obtained from 110 patients with advanced OSCC treated at Kumamoto University Hospital between October 2003 and October 2011. All patients were preoperatively treated with concurrent chemoradiotherapy (CRT) including a total radiation dose of 30 Gy followed by curative surgery in a phase II study^19,20^. The treatment strategies for patients with OSCC were planned according to the Clinical Practice Guidelines for Oral Cancer^21^. All tumors were staged according to the eighth edition of the TNM classification of the American Joint Committee on Cancer (2017). The degree of differentiation was determined according to the grade classification of the World Health Organization. This study was conducted in accordance with the guidelines of the Ethics Committee of Kumamoto University (project identification code: SENSHIN No. 2389 and RINRI No. 1427). Informed consent was obtained from all patients before biopsy and surgery based on the guidelines of Kumamoto University (SENSHIN No. 2389). This study was a retrospective analysis, which did not require individual consent; however, patients were provided an opportunity to refuse participation via an opt-out format (RINRI No. 1427). Tissue samples derived from biopsy specimens obtained before preoperative CRT were used for immunohistochemical analyses. The samples were fixed in 10% formalin and embedded in paraffin.

### Immunohistochemical staining and histopathological evaluation

It is generally thought that p-Nrf2 translocated to the nucleus plays an important function as a transcription factor in cells^22^; therefore, we decided to investigate the clinicopathological significance of Nrf2 using p-Nrf2-specific antibodies. Immunohistochemical staining was performed as previously described^23^. Anti-human phosphorylated Nrf2 (p-Nrf2, Ser40; EP1809Y, 1:100; Abcam, Cambridge, UK), anti-human Ki-67 antibodies (1:100; Dako, Glostrup, Denmark) were used. The expression of p-Nrf2 and Ki-67 was quantified using ImageJ version 1.53 (NIH, Bethesda, Maryland, USA). Using the specimens obtained from surgery, the histological responses to CRT were graded according to the criteria proposed by Shimasoto et al.^24^ as follows: grade I, tumor structures are not destroyed; grade IIa, the destruction of the tumor structure is mild (i.e., “viable tumor cells” are frequently observed); grade IIb, the destruction of the tumor structure is severe (i.e., “viable tumor cells” are sparse); grade III, nonviable tumor cells are present; and grade IV, no tumor cells remain.

### Assessment of immunohistochemical staining

p-Nrf2 expression was determined by counting the number of cancer cells with positive p-Nrf2–stained nuclei. Normal human placental tissue samples served as positive controls, and primary oral cancer tissues that were treated similarly to the samples excluding the replacement of the primary antibody with universal negative control anti-rabbit antibody (Dako) were used as negative controls. Three independent observers (KK, NT, and MH) interpreted the immunohistochemical data in a blinded manner. For each specimen, p-Nrf2 expression was assessed using the staining intensity and proportion of positive cells in high-power fields (×20 objective and ×10 ocular). Nuclear p-Nrf2 expression was quantified using a four-value intensity score (0, 1+, 2+, or 3+) in addition to the extent of reactivity expressed as a percentage (0–100%). The p-Nrf2 immunohistochemical expression score was determined by multiplying the intensity score and extent of reactivity (range, 0–300). High p-Nrf2 expression was defined as a score exceeding 80, which represents the median expression for OSCCs evaluated using whole-biopsy tissue sections. Immunohistochemical staining scores that were inconsistent between examiners were finally settled by consensus.

### Irradiation

Irradiation was administered at doses of 2, 6, and 10 Gy using a 150-KVp X-ray generator (Model MBR-1520R; Hitachi, Tokyo, Japan) with total filtration using a 0.5-mm aluminum plus 0.1-mm copper filter. The dose rate as measured by a thimble ionization chamber (IC 17A, Far West Technology, Goleta, CA, USA) was 1.01 Gy/min.

### Western blot analysis

Whole-cell and nuclear protein (Minute™ Cytoplasmic & Nuclear Extraction Kits; Invent Biotechnologies, Eden Prairie, MN, USA) was separated using 7.5% or 10.0% SDS-PAGE, transferred onto nitrocellulose membranes, and probed with antibodies against Nrf2 (1:15,000; Abcam), p-Nrf2 (Ser40) (1:15,000; Abcam), Keap1 (1:1000; Cell Signaling Technology, Danvers, MA, USA), phosphorylated p62 (phospho-p62, Ser351, 1:1000; Medical & Biological Laboratories Co., LTD, Woburn, MA, USA), STAT3 (1:1000; Cell Signaling Technology), phosphorylated STAT3 (phospho-STAT3, Thy705, 1:500; Cell Signaling Technology), Lamin B1 (1:10,000; Abcam), and β-actin (1:10,000; Sigma-Aldrich, St. Louis, MO, USA). After overnight incubation at 4 °C, the membranes were washed and incubated with appropriate horseradish peroxidase-conjugated secondary antibodies and visualized using an ECL prime detection kit (GE Healthcare, Buckinghamshire, UK).

### Modified high-density survival (HDS) assay

The modified HDS assay was performed according to the method of Kuwahara et al.^25^. Exponentially growing cells (5 × 10^5^) were seeded in a 60-mm tissue culture dish (Asahi Techno Glass, Shizuoka, Japan) and incubated in DMEM containing 1% FBS for 48 h. The cells were then exposed to 2, 4, 6, or 10 Gy of radiation. After 72 h of incubation, 10% of the cells in each flask were seeded in a new 60-mm culture dish and incubated for a further 72 h. Finally, the total number of cells in each culture dish was counted via the Trypan blue dye exclusion test, and the survival of the cells was plotted.

### Cell proliferation assay

To assess normal proliferation, OSCC and CRR cells were quantified every 24 h using the Cell Counting Kit-8 (Dojindo, Kumamoto, Japan).

### RNA isolation and real-time quantitative polymerase chain reaction (RT-qPCR)

Total RNA was isolated from the treated cells using a mirVana™ miRNA Isolation Kit (Life Technologies, Palo Alto, CA, USA). Total RNA was then reverse-transcribed to cDNA using a ReverTra qPCR RT Kit (Toyobo, Osaka, Japan). Each reaction was run using Thunderbird SYBR qPCR Mix (Toyobo) on a Light Cycler 1.5 (Roche, Indianapolis, IN, USA). The comparative Ct method was used to determine the fold changes in the expression using glyceraldehyde-3-phosphate dehydrogenase (GAPDH) as a control. Each sample was run in triplicate. Primers were used for Nrf2 (forward, 5′-AGTGGATCTGCCAACTACTC-3′; reverse, 5′-CATCTACAAACGGGAATGTCTG-3′), malic enzyme 1 (ME1; forward, 5′-CTGCCTGTCATTCTGGATGT-3′; reverse, 5′-ACCTCTTACTCTTCTCTGCC-3′), glutamate-cysteine ligase catalytic subunit (GCLC; forward, 5′-TGAAGGGACACCAGGACAGCC-3′; reverse, 5′-GCAGTGTGAACCCAGGACAGC-3′); glutamate-cysteine ligase modifier subunit (GCLM; forward, 5′-AATCTTGCCTCCTGCTGTGTGA-3′; reverse, 5′-TGCGCTTGAATGTCAGGAATGC-3′), and GAPDH (forward, 5′-CAACAGCCTCAAGATCATCAGC-3′; reverse, 5′-TTCTAGACGGCAGGTCAGGTC-3′). The cycling conditions consisted of initial denaturation at 98 °C for 5 min followed by 45 cycles at 98 °C for 15 s, 58 °C for 30 s, and 72 °C for 60 s. These experiments were performed in triplicate.

### Transfection with small interfering RNA (siRNA)

OSCC and CRR cells were used in this experiment. Twenty-four hours before siRNA transfection, the cells were diluted in fresh medium without antibiotics and transferred to 60-mm culture dishes. The cells were grown and transfected with Nrf2-specific siRNA and Stealth™ RNAi Universal negative control (40 nM, Stealth siRNA, Invitrogen, Carlsbad, CA, USA) using Lipofectamine RNAi MAX (Invitrogen), as described in the manufacturer’s instructions. The siRNA sequences were as follows: Nrf2-1 sense, 5′-CCA ACC AGU UGA CAG UGA ACU CAU U-3′; Nrf2-1 antisense, 5′-AAU GAG UUC ACU GUC AAC UGG UUG G-3′; Nrf2-2 sense, 5′-CAA ACU GAC AGA AGU UGA CAA UUA U-3′; and Nrf2-2 antisense, 5′-AUA AUU GUC AAC UUC UGU CAG UUU G-3′. The cells were harvested 48 h after transfection for in vitro assays.

### Seahorse XF24 metabolic flux analysis

The glycolysis stress test was performed using a Seahorse XF24 analyzer (Seahorse Biosciences, Santa Clara, CA, USA). The oxygen consumption rate (OCR) and extracellular acidification rate (ECAR) were measured before and after treatment with glucose (25 mM) and the glycolysis inhibitor 2-DG (100 µM; Wako, Osaka, Japan). Exponentially growing OSCC cells and their respective CRR clones were seeded at 5 × 10^4^ cells/well in a 24-well cell culture XF microplate (Seahorse Biosciences) and allowed to adhere overnight with DMEM for XF. Cells were then washed with assay medium (unbuffered DMEM with no glucose, pH 7.4) before incubation with assay medium without glucose (pH 7.4, 675 µl) for 1 h at 37 °C in a CO_2_-free incubator. Four baseline OCR and ECAR measurements were performed over 28 min before the injection of glucose. OCR and ECAR measurements were also performed over 32 min following the injection of glucose. Then, OCR and ECAR measurements were performed over 32 min following the injection of 2-DG.

### Oxidized glutathione (GSSG)/reduced glutathione (GSH) quantification assay

The cells were irradiated or transfected with siRNA. Then, the cells were harvested 24 h after irradiation or 48 h after transfection. GSSG/GSH levels were quantified using a GSSG/GSH Quantification Kit (Dojindo) according to the manufacturer’s instructions.

### Cellular ROS/superoxide detection assay

First, HSC-2-WT and HSC-2-R cells were incubated for 48 h after siRNA transfection. Second, the cells (2 × 10^3^) were seeded onto 96-well plates (Asahi Techno Glass) and incubated in DMEM containing 1% FBS for 24 h. Then, the cells were exposed to 10 Gy of radiation. After 24 h of incubation, the appearance of ROS/superoxide in the cells was detected using a Cellular ROS/Superoxide Detection Assay Kit (Abcam). The observations were performed using a FilterMAX F5 microplate reader (Molecular Devices, San Jose, CA, USA). The ROS/superoxide assay protocol is based on two fluorescent dyes: Oxidative Stress Detection Reagent (Ex/Em 490/525 nm) for detecting total ROS and Superoxide Detection Reagent (Ex/Em 550/620 nm).

### Statistical analysis

The chi-square test was used to determine the associations of the p-Nrf2 expression status with clinical parameters. Survival analysis was performed using the Kaplan–Meier method, and the log-rank test was used to determine the correlation between p-Nrf2 expression and patient survival. Multivariate survival analyses were performed using the Cox regression model to study the effects of p-Nrf2 expression on overall survival (OS) and disease-free survival (DFS). The differences in values between two groups were statistically analyzed using Student’s *t* test, whereas the differences in values among three or more groups were analyzed using one-way analysis of variance with the Bonferroni–Dunn test. All *p* values were based on two-tailed statistical analyses, and *p* < 0.05 indicated statistical significance. All statistical analyses were performed using the JMP 9 software program (SAS Institute Inc., Cary, NC, USA).

## Results

### p-Nrf2 is overexpressed in CRR cells in vitro and in vivo

**Fig. 1:**
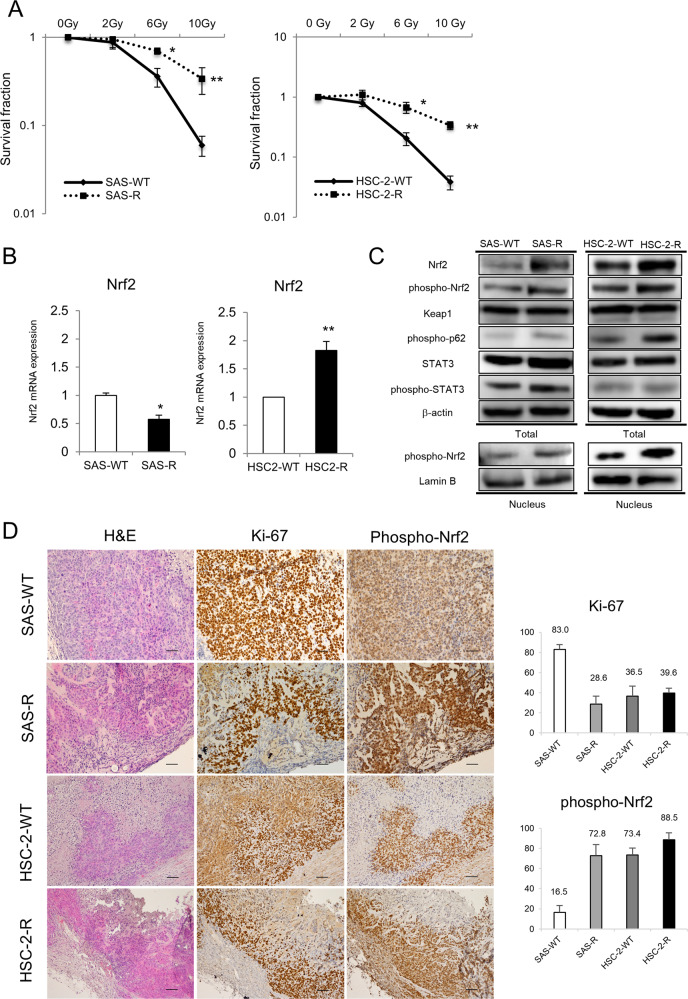
Nuclear factor erythroid 2-related factor 2 (Nrf2) activation is increased in clinically relevant radioresistant (CRR) cells. **A** The surviving fractions of SAS cells, HSC-2 cells, and their respective clinically relevant radioresistant (CRR) clones after exposure to 2, 6, and 10 Gy of X-ray radiation were evaluated using a modified high-density survival assay. *p < 0.05 and **p < 0.01. **B** The mRNA levels of Nrf2 in SAS cells, HSC-2 cells, and their respective CRR clones under normal conditions were analyzed by real-time quantitative polymerase chain reaction. The results are presented as the mean ± SD of three independent experiments. *p < 0.05 and **p < 0.01. **C** The protein expression of Nrf2, phosphorylated Nrf2 (phospho-Nrf2), Keap1, phosphorylated p62 (phospho-p62), STAT3, and phosphorylated STAT3 (phospho-STAT3) in SAS, HSC-2, and CRR cells under normal conditions. Whole-cell and nuclear protein was prepared, and the expression of Nrf2, phospho-Nrf2, Keap1, phospho-p62, STAT3, and phospho-STAT3 was examined via Western blotting. **D** Representative microscopic images of H&E and immunohistochemical staining of phospho-Nrf2 and Ki-67 in oral squamous cell carcinoma tissues excised on day 21 after xenotransplantation. Original magnification, ×100; bar, 100 µm. Phospho-Nrf2 and Ki-67 were quantified using ImageJ software.

First, to confirm whether SAS-R and HSC-2-R cells acquired the CRR phenotype, OSCC and CRR cells were irradiated as described by Kuwahara et al.^25^. The survival rate of both SAS-R and HSC-2-R cells was significantly higher than that of their corresponding parental cells (Fig. 1A). Second, we confirmed the expression of Nrf2, p-Nrf2, and Nrf2 activation-related molecules (Keap1, p62, and STAT3) in OSCC and CRR cells using RT-qPCR and Western blot. Third, we examined the expression pattern of p-Nrf2 in xenografted tumors in mice via immunohistochemistry. Although radioresistant SAS-R cells had significantly higher levels of Nrf2 and p-Nrf2 protein in the cytoplasm and nucleus, the mRNA level of Nrf2 was lower in SAS-R cells than in parental cells (Fig. 1B, C). In contrast, in HSC-2-R cells, overexpression of Nrf2 and p-Nrf2 was observed in both the mRNA and protein levels compared to HSC-2 cells (Fig. 1B, C). Increased expression of Keap1 and phosphorylated p62, which are positive regulators of p-Nrf2, as well as phosphorylation of STAT3, which is thought to be involved in regulating the expression of these molecules in CRR cells were observed^10^. Furthermore, we showed that Nrf2 protein was overexpressed in many OSCC cell lines (Supplementary Fig. S1). Additionally, in the in vivo mouse xenograft model, the expression of p-Nrf2, the active form of Nrf2, was significantly increased in CRR cells; however, there was no difference in proliferative activity (Fig. 1D). Moreover, we confirmed that p-Nrf2 expression in irradiated (10 Gy) CRR cells was higher than that in non-irradiated cells (data not shown).

### Nrf2 suppression sensitized OSCC and CRR cells to radiation

**Fig. 2:**
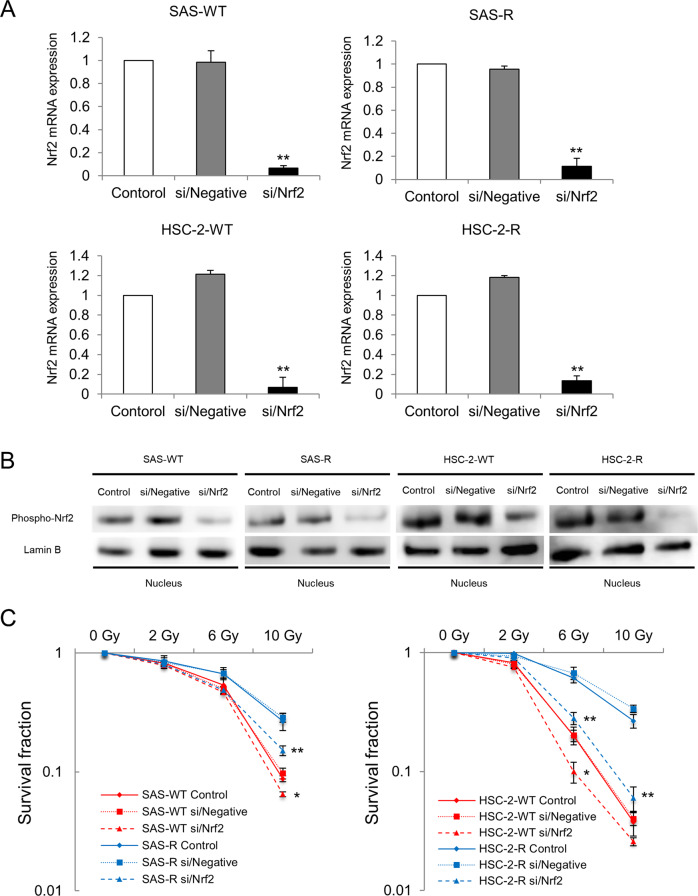
Downregulation of nuclear factor erythroid 2-related factor 2 (Nrf2) using small interfering RNA (siRNA) increases the radiosensitivity of radioresistant oral squamous cell carcinoma (OSCC) cells. **A** The mRNA levels of Nrf2 in SAS cells, HSC-2 cells, and their respective clinically relevant radioresistant (CRR) clones transfected with control, si/Negative, or si/Nrf2 transfection. At 48 h after transfection, total RNA was extracted, and Nrf2 mRNA expression as analyzed by real-time quantitative polymerase chain reaction. The results are presented as the mean ± SD of three independent experiments. **B** Nrf2 protein expression in SAS, HSC-2, and CRR cells following control, si/Negative, or si/Nrf2 transfection. At 48 h after transfection, the nuclear fraction was prepared, and then phosphorylated Nrf2 (phospho-Nrf2) protein expression was examined via Western blotting. **C** The effects of Nrf2 downregulation on radiosensitivity in OSCC and CRR cells. At 48 h after control, si/Negative, or si/Nrf2 transfection, cells were exposed to 2, 6, or 10 Gy of X-ray radiation. Then, the surviving fraction was determined using a modified high-density survival assay. *p < 0.05 and **p < 0.01.

We investigated whether Nrf2 downregulation affects radiosensitivity in irradiated OSCC and CRR cells. As the data obtained from all experiments were similar between the Nrf2-1 and Nrf2-2 siRNA constructs, we only presented the data for Nrf2-1 for the subsequent experiments involving the suppression of Nrf2 (Supplementary Fig. S2). First, we confirmed the inhibitory effect of siRNA on Nrf2 expression in OSCC and CRR cells at the mRNA and nuclear protein level (Fig. 2A, B). Second, to determine whether the downregulation of Nrf2 affects the radiosensitivity of OSCC and CRR cells, we examined their radiosensitivity after suppressing Nrf2 expression using a modified HDS assay, observing that Nrf2 suppression resulted in a significant decrease in the surviving fractions of irradiated OSCC and CRR cells (Fig. 2C).

### Evaluation of glycolytic metabolism in OSCC and CRR cells via Seahorse XF metabolic flux analysis

**Fig. 3:**
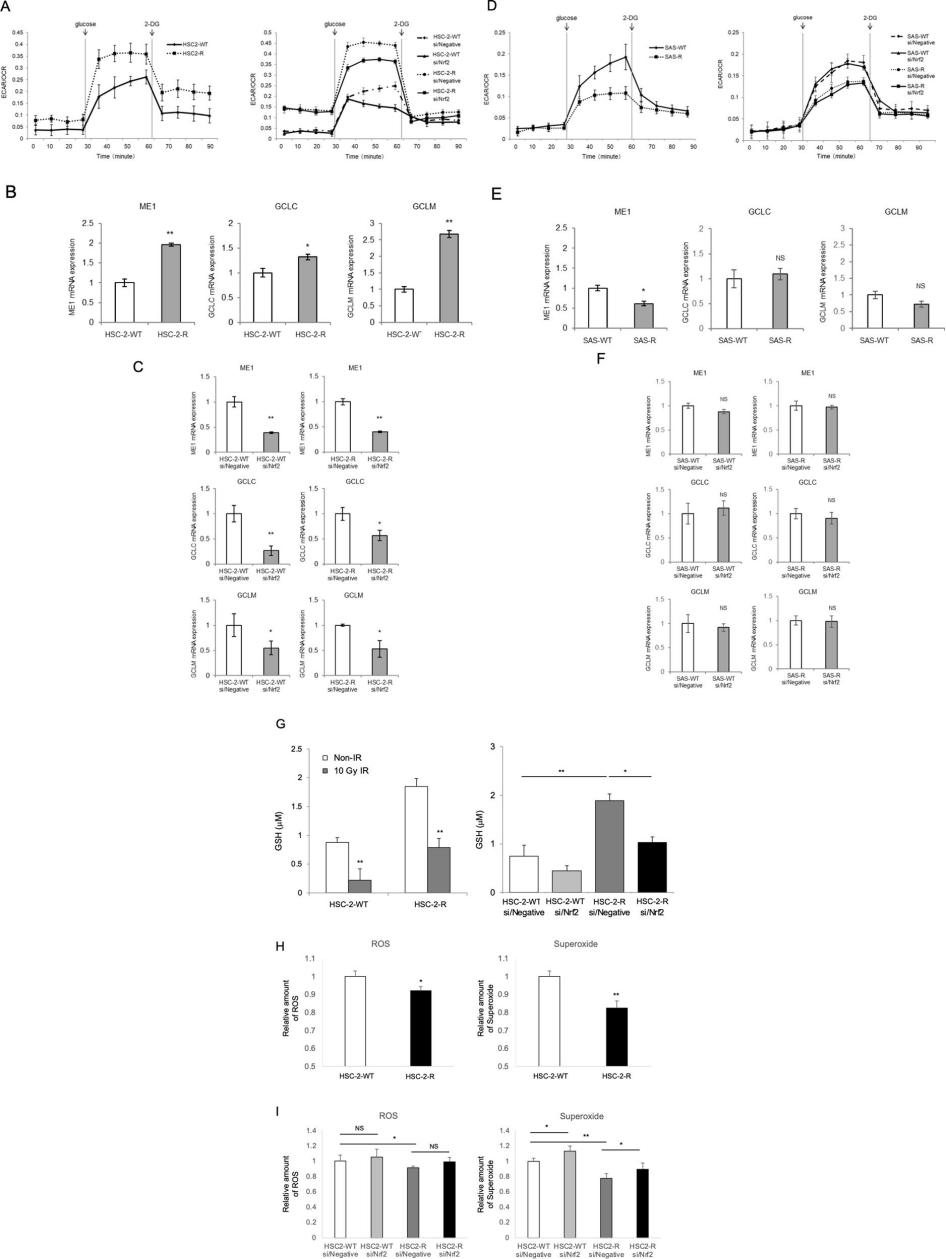
Nuclear factor erythroid 2-related factor 2 (Nrf2) scavenges reactive oxygen species (ROS) via glutathione (GSH) depending on glycolytic metabolism in radioresistant oral squamous cell carcinoma cells (OSCC). **A**, **D** The glycolysis stress test was performed using a Seahorse XF24 analyzer. **A** The oxygen consumption rate (OCR) and extracellular acidification rate (ECAR) were measured using HSC-2-WT cells and HSC-2-R cells before and after treatment with glucose and 2-deoxyglucose (2-DG). At 48 h after transfection, HSC-2-WT and HSC-2-R cells were seeded, after which the glycolysis stress test was performed. **D** The glycolysis stress test was performed using SAS-WT cells and SAS-R cells in the same way as in **A**. **B**, **C**, **E**, **F** The mRNA levels of malic enzyme 1 (ME1) and glutamate-cysteine ligase catalytic subunit (GCLC)/glutamate-cysteine ligase modifier subunit (GCLM) were investigated using real-time quantitative polymerase chain reaction (RT-qPCR). The results are presented as the mean ± SD of three independent experiments. **B**, **E** The mRNA levels of ME1 and GCLC/GCLM in OSCC and CRRR cells under normal conditions. **C**, **F** The mRNA levels of ME1 and GCLC/GCLM in OSCC cells and CRR cells transfected with si/Negative or si/Nrf2. At 48 h after transfection, total RNA was extracted, and the mRNA expression of Nrf2 was analyzed by RT-qPCR. **G**–**I** Nrf2-mediated-antioxidant capacity was examined using the GSSG/GSH quantification assay. **G** GSH production in HSC-2-WT and HSC-2-R cells 24 h after exposure to 10 Gy of radiation. GSH production in HSC-2-WT and HSC-2-R cells at 48 h after transfection with si/Negative or si/Nrf2. **H** At 24 h after irradiation, ROS/superoxide levels were examined in cells using a Cellular ROS/Superoxide Detection Assay Kit. **I** At 24 h after irradiation, ROS/superoxide levels were examined in cells transfected with si/Negative or si/Nrf2. The ROS/superoxide assay protocol is based on two fluorescent dyes: Oxidative Stress Detection Reagent (Ex/Em 490/525 nm) for the detection of total ROS and Superoxide Detection Reagent (Ex/Em 550/620 nm). *p < 0.05 and **p < 0.01.

Recent studies have revealed that the Nrf2-mediated metabolic pathway is involved in cancer radioresistance^26^. Therefore, we examined glycolytic metabolism in OSCC cells and their respective CRR cells. In HSC-2 with a nonfunctional *TP53* mutation^27^ and its CRR cell line HSC-2-R, glycolytic activity was higher in HSC-2-R cells than in HSC-2-WT cells. However, glycolysis decreased in HSC-2-WT and HSC-2-R cells through Nrf2 suppression by siRNA (Fig. 3A). Further, we investigated changes in expression levels of genes associated with glycolysis and the TCA cycle using HSC-2-WT and HSC-2-R cells. The results showed that HSC-2-R cells strongly expressed ME1 and GCLC/GCLM, which synthesize pyruvate and glutathione, respectively (Fig. 3B). In contrast, ME1 and GCLC/GCLM mRNA expression decreased significantly under Nrf2-specific siRNA treatment conditions (Fig. 3C). In SAS-WT with a functional *TP53* mutation^28,29^ and its CRR cell line SAS-R, glycolytic activity was lower in CRR cells than in HSC-2-WT and HSC-2-R cells. The change in glycolytic activity was small compared to HSC-2-WT and HSC-2-R under Nrf2 knockdown conditions (Fig. 3D). Although ME1 mRNA expression was significantly lower in SAS-R cells than in SAS cells, no significant expression changes in GCLC/GCLM were observed in SAS-R cells compared to SAS cells (Fig. 3E). Furthermore, there was no significant change in ME1 and GCLC/GCLM expression under Nrf2 knockdown conditions in SAS cells (Fig. 3F). Since *TP53* is the most commonly mutated gene (approximately 70–80%) in OSCC^30^, we performed further in vitro experiments focused on HSC-2-WT and HSC-2-R cells.

### Effect of Nrf2 on the antioxidant capacity of HSC-2-R cells

We investigated whether Nrf2 modulates oxidative stress via glutathione in HSC-2-R cells. First, we examined the antioxidant capacity using the GSSG/GSH quantification assay. HSC-2-R cells had significantly higher GSH levels under normal conditions and under oxidative stress. Meanwhile, GSH levels were strongly reduced upon Nrf2 suppression in HSC-2-WT and HSC-2-R cells (Fig. 3G). Similarly, ROS/superoxide levels were reduced in HSC-2-R cells after exposure to 10 Gy of radiation (Fig. 3H). Moreover, ROS/superoxide levels were increased in HSC-2-WT and HSC-2-R cells following Nrf2 suppression using siRNA (Fig. 3I). These results suggest that HSC-2-WT and HSC-2-R cells scavenge ROS via glutathione under activated Nrf2.

### Clinical significance of p-Nrf2 expression in tumors of patients with OSCC

**Fig. 4:**
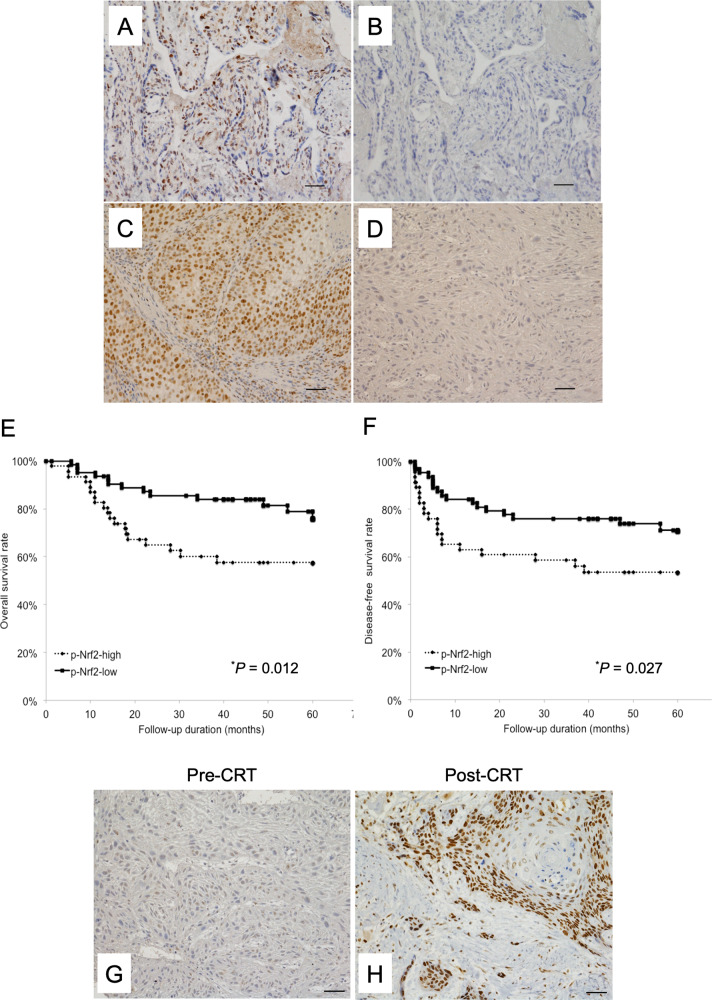
High expression of phosphorylated Nrf2 (p-Nrf2) is associated with overall survival and disease-free survival in patients with oral squamous cell carcinoma (OSCC). **A**–**D** Representative images of immunohistochemical staining of p-Nrf2 are presented according to its expression (original magnification, ×200). Tissues were immunohistochemically stained using antibodies specific for p-Nrf2 (Ser40). **A** Human normal placental tissue (positive control). **B** Human normal placental tissue (negative control). **C** A patient exhibiting high-intensity staining for p-Nrf2. **D** A patient with low-intensity staining for p-Nrf2. Bar, 50 µm. **E**, **F** The Kaplan–Meier curves of 110 patients with OSCC based on p-Nrf2 expression. Patients with OSCC were divided into two groups based on their p-Nrf2 expression status (p-Nrf2-low vs. p-Nrf2-high). **E** Overall survival. **F** Disease-free survival. *p < 0.05. **G**, **H** OSCC tissue samples stained with p-Nrf2 using immunohistochemistry. **G** There was no expression of p-Nrf2 in biopsy samples acquired before chemoradiotherapy (CRT). **H** Cells resistant to CRT express p-Nrf2 in the primary lesion. Original magnification, ×200; bar, 50 µm.

To further identify the special role of functional Nrf2 in OSCC tissue based on our in vitro data, we examined p-Nrf2 expression in the biopsy specimens of 110 patients with OSCC via immunohistochemical staining. Representative results are presented in Fig. 4A–D. p-Nrf2 immunoreactivity was observed to varying degrees in the nuclei of cancer cells. The clinicopathological characteristics of the study population are summarized in Table 1. The patients were divided into two groups (p-Nrf2-high and p-Nrf2-low) according to the expression of p-Nrf2 as described in the Materials and Methods. The frequency of p-Nrf2 positivity was significantly higher in patients with a poor pathological response to preoperative CRT (*p* = 0.010, Table 1). In contrast, there were no significant differences in several clinicopathological characteristics according to the p-Nrf2 status. The 5-year OS rate of patients with p-Nrf2-high tumors was significantly lower than that of patients with p-Nrf2-low tumors (*p* = 0.018, Fig. 4E). Moreover, positive expression of p-Nrf2 was associated with poorer 5-year DFS rates in patients with OSCC (*p* = 0.039, Fig. 4F). Furthermore, although the percentage of p-Nrf2-high tumors before CRT was 41.8%, almost all residual OSCC cells after preoperative CRT displayed strong Nrf2 expression (Fig. 4G, H). On multivariate analysis of OS and DFS using the Cox proportional hazards regression model, pN-stage, the pathological response to preoperative CRT and p-Nrf2 expression were significant prognostic factors (Table 2).

**Table 1:** Clinical characteristics of patients (*n* = 110) categorized according to the p-Nrf2 expression level.

Characteristics	Total	p-Nrf2 status		*p* value
		Low *n* (%)	High *n* (%)	
	110	64 (58.2)	46 (41.8)	
Age, years				
Median	67.0	67.4	66.5	
Range	30–87	30–85	33–87	
≤65	45	25 (55.6)	20 (44.4)	0.642
>65	65	39 (60.0)	26 (40.0)	
Sex				0.528
Male	66	40 (60.6)	26 (39.4)	
Female	44	24 (54.5)	20 (45.5)	
Primary site				0.742
Tongue	34	17 (50.0)	17 (50.0)	
Mandible	25	16 (64.0)	9 (36.0)	
Maxilla	22	13 (59.1)	9 (40.9)	
Oral floor	13	9 (69.2)	4 (30.8)	
Buccal mucosa	16	9 (56.3)	7 (43.7)	
pT-stage				0.951
T1, T2	42	24 (57.1)	18 (42.9)	
T3	28	17 (60.7)	11 (39.3)	
T4	40	23 (57.5)	17 (42.5)	
pN-stage				0.977
*N* = 0	62	36 (58.1)	26 (41.9)	
*N* ≥ 1	48	28 (58.3)	20 (41.7)	
Clinical stage				0.541
II	13	7 (53.8)	6 (46.2)	
III	30	20 (66.7)	10 (33.3)	
IV	67	37 (55.2)	30 (44.8)	
Differentiation				0.549
Well	89	53 (59.6)	36 (40.4)	
Moderate	21	11 (52.4)	10 (47.6)	
Mode of invasion				0.967
Grade I	2	2 (100.0)	2 (0.0)	
Grade II	18	10 (55.6)	8 (44.4)	
Grade III	62	35 (56.5)	27 (43.5)	
Grade IVc, IVd	28	17 (60.7)	11 (39.3)	
Pathological response				0.010*
Grade = 0, I, IIa	21	7 (33.3)	14 (66.7)	
Grade ≧ IIb	89	57 (64.0)	32 (36.0)	

The chi-square test was used to examine the relationship between the p-Nrf2 expression and the clinicopathological factors.

*p < 0.05.

**Table 2:** Multivariate Cox proportional hazards regression analysis of overall and disease-free survival in patients with oral squamous cell carcinoma (*n* = 110).

		OS		DFS	
Variables	Assigned score	Hazard ratio (95% CI)	*p* value	Hazard ratio (95% CI)	*p* value
Age, years					
≤65	0	0.852 (0.354–2.024)	0.716	1.200 (0.556–2.614)	0.642
>65	1				
Sex					
Male	0	0.792 (0.352–1.747)	0.564	0.873 (0.419–1.791)	0.712
Female	1				
Primary site					
Tongue	1	0.922 (0.710–1.181)	0.527	1.065 (0.421–2.614)	0.892
Mandible	2				
Maxilla	3				
Oral floor	4				
Buccal mucosa	5				
pT-stage					
T1, T2	1	1.098 (0.716–1.700)	0.668	2.263 (0.710–7.492)	0.168
T3	2				
T4	3				
pN-stage					
*N* = 0	0	3.526 (1.643–8.030)	0.001**	2.822 (1.421–5.781)	0.003**
*N* ≥ 1	1				
Differentiation					
Well	0	1.015 (0.424–2.250)	0.923	1.035 (0.445–2.194)	0.933
Moderate	1				
Pathological response					
Grade = 0, I, IIa	0	0.284 (0.123–0.659)	0.004**	0.331 (0.158–0.711)	0.005**
Grade ≧ IIb	1				
p-Nrf2 status					
Low	0	2.224 (1.070–4.747)	0.032*	2.039 (1.041–4.041)	0.038*
High	1				

*CI* confidence interval, *OS* overall survival, *DFS* disease-free survival.

*p < 0.05; **p < 0.01.

## Discussion

CRR cells were established by Kuwahara et al. to understand the mechanism of radioresistance^18^. The findings of this study using the newly established HSC-2-R cell line support previous studies by revealing that CRR cells should be considered as a suitable research resource for elucidating mechanisms of radioresistance in cancer cells. To our knowledge, no other reports have identified the molecular mechanisms underlying radioresistance in OSCC using CRR cells. Different from previous studies investigating OSCC radioresistance, this is the first study using two CRR cell lines to elucidate the mechanism of radioresistance in OSCC.

In this study, both Nrf2 and its activated form, p-Nrf2, were overexpressed in CRR clones. However, the changes in the expression of Nrf2 mRNA were inconsistent. These results might be attributed partly to Nrf2’s negative feedback, especially in SAS-R cells. We previously reported that Nrf2 phosphorylation in OSCC after irradiation depends on the Keap1/Nrf2 system, which may be regulated by IL-6-STAT3 pathway^10^. Accordingly, elevated phosphorylation of STAT3 was observed in both CRR cell lines. Moreover, we found that Keap1, which is a positive regulator of Nrf2, and phosphorylated p62^31,32^ were upregulated in CRR cells. These results suggest that the stabilization of Nrf2 following activation of the STAT3 pathway, which is required for nuclear translocation of p-Nrf2, plays a role in the elevated expression of Nrf2 and p-Nrf2 in CRR cells.

Nrf2 is a critical component of the defensive antioxidant response mechanism that protects normal cells from damaging oxidative conditions^4,33^. However, there is increasing evidence that Nrf2 and its downstream target genes are overexpressed in many types of human cancer^34^ and that it may play an important role in the development and progression of cancer^8,34,35^. We similarly observed increased nuclear localization of p-Nrf2 (which is indicative of its activation) in OSCC tumors compared to that in normal and dysplastic tissues (Supplementary Fig. S3). Additionally, we Nrf2 expression and activation in are higher CRR cells than in the parental OSCC cells. Moreover, suppressing Nrf2 sensitizes OSCC and CRR cells to radiation. These results suggest that malignant phenotypes including radioresistance in OSCC cells can be regulated by Nrf2 antioxidant pathway, a notion supported by evidence illustrating that Nrf2-mediated antioxidation plays an important role in the development and progression of malignant tumors.

Recently, we demonstrated that OSCC cells exhibit decreased radiosensitivity when ROS generation is suppressed by the Nrf2 antioxidant system^10^. The enhanced Nrf2-mediated-antioxidant response that scavenges ROS in cancer cells allows these cells to escape the damaging effects of radiotherapy^1,3,36,37^. Indeed, Nrf2 is a key transcriptional regulator of genes encoding numerous cytoprotective enzymes in response to oxidative stress^1^. In this study, we elucidated that Nrf2 in radioresistant HSC-2-R cells controlled the expression of ME1 and GCLC/GCLM as well as regulated antioxidant capacity, thereby suppressing glutathione levels. The ability to scavenge ROS through the enhancement of GSH levels confers radioresistance to cancer cells^38,39^. Additionally, the nuclear accumulation of Nrf2 enables it to promote metabolic activities involving glucose metabolism^14^. Moreover, Nakashima et al. reported that ME1 expression is associated with glycolysis in human OSCC^40^. Therefore, it is possible that the metabolic reprogramming leading to the activation of glycolysis and glutamine metabolism is associated with radioresistance through the enhancement of Nrf2-mediated antioxidation involved in the inhibition of ROS generation.

Many noteworthy findings were obtained for Nrf2 in this study. In SAS-WT and SAS-R cells, no significant Nrf2-dependent changes in glycolytic metabolism were observed compared to HSC-2-WT and HSC-2-R cells. In a recent study, Harami et al. reported that mutant p53-expressed tumors were often associated with upregulation of glycolytic enzymes^41^. Moreover, it has been reported that the nonfunctional mutant *TP53* promotes the uptake of pyruvate under glucose starvation, thereby protecting cancer cells against oxidative stress in malignant melanomas^42^. In contrast, Faraonio et al. reported that p53 activation suppresses the Nrf2-dependent antioxidant stress response^43^. As already reported, SAS has mutations in *TP53* but no loss of function^28,29,44^. Considering the abovementioned findings, the results of this study suggest that there is a difference in Nrf2-dependent metabolic changes depending on the status of *TP53* mutation between SAS cells and HSC-2 cells^27,29^. However, further studies on the role of p53 and Nrf2 in OSCC radioresistance are needed.

Strong nuclear Nrf2 expression in cancer cells is also correlated with worse clinical features^45–48^. Notably, Kawasaki et al. reported that nuclear Nrf2 expression was significantly associated with unfavorable responses to CRT in patients with esophageal squamous cell carcinoma^45^. Our data suggest that p-Nrf2 contributes to resistance to chemotherapy and/or radiotherapy in OSCC cells. As no studies (to our knowledge) examined the relationship between Nrf2 expression and prognosis of patients with OSCC who specifically underwent preoperative 5-FU–based CRT, our study is the first to demonstrate that high p-Nrf2 expression is significantly correlated not a poor response to CRT and shorter DFS and OS in patients with OSCC. Moreover, as observed in other malignancies, our data indicated that p-Nrf2 expression is an independent prognostic factor in patients with OSCC. Moreover, preoperative CRT dramatically increased the percentage of cells with strong Nrf2 expression. These data suggest that the Nrf2 antioxidant pathway contributes to both intrinsic and acquired chemoradioresistance in clinical OSCC.

Some study limitations must be noted. A major limitation is the use of one CRR clone with a nonfunctional mutation of *TP53*, and a paucity of data for radiosensitivity and survival under inhibition of Nrf2 using in vivo mouse models. To address this limitation, we are currently establishing another CRR clone. Further studies must confirm the effects of combination therapy under Nrf2 inhibition in multiple radioresistant OSCC cells using in vivo models.

In conclusion, we demonstrated for the first time that Nrf2 controlled resistance to radiation by antioxidant capacity accompanied with metabolic modulation in OSCC cells and that p-Nrf2 expression was closely related to the outcome of CRT and associated with patient prognosis in OSCC following preoperative CRT. Our data indicate that targeting the Nrf2 antioxidant pathway to overcome chemoradioresistance would enhance the responses to treatment in patients with refractory OSCC, thereby improving survival rates.

## Supplementary information

Supplementary Figure S1

Supplementary Figure S2

Supplementary Figure S3

Supplemental Figure Legends

## Data Availability

All data generated or analyzed during this study are included in this published article.
