# Supplementary figures and images for: The antioxidative stress regulator Nrf2 potentiates radioresistance of oral squamous cell carcinoma accompanied with metabolic modulation
Source: Lab Invest. 2022 Apr 12;102(8):896–907. doi: 10.1038/s41374-022-00776-w (PMC9309095; doi:10.1038/s41374-022-00776-w)

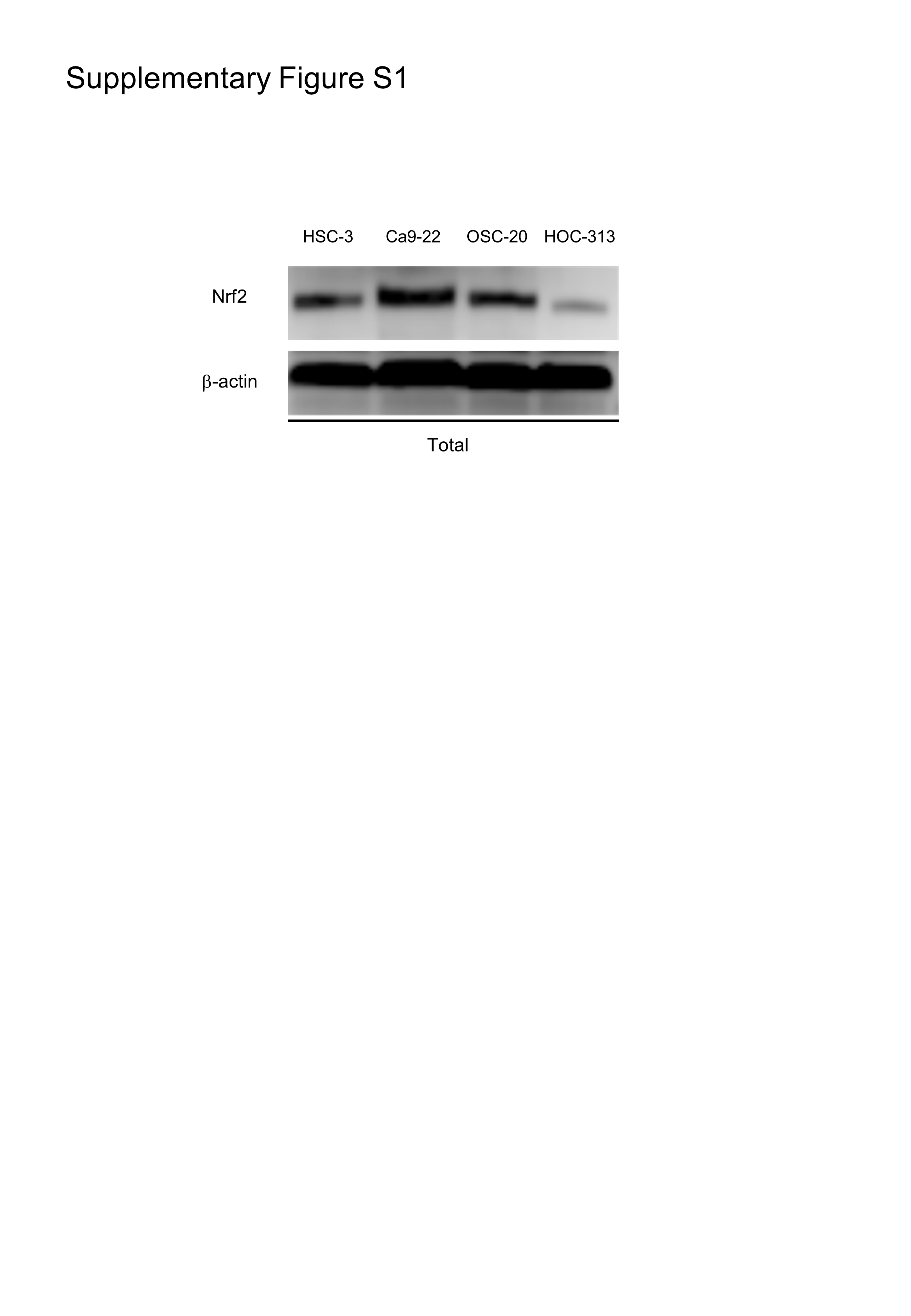

Supplement: Supplementary file 1 — Supplementary Figure S1 [file 41374_2022_776_MOESM1_ESM.tif]

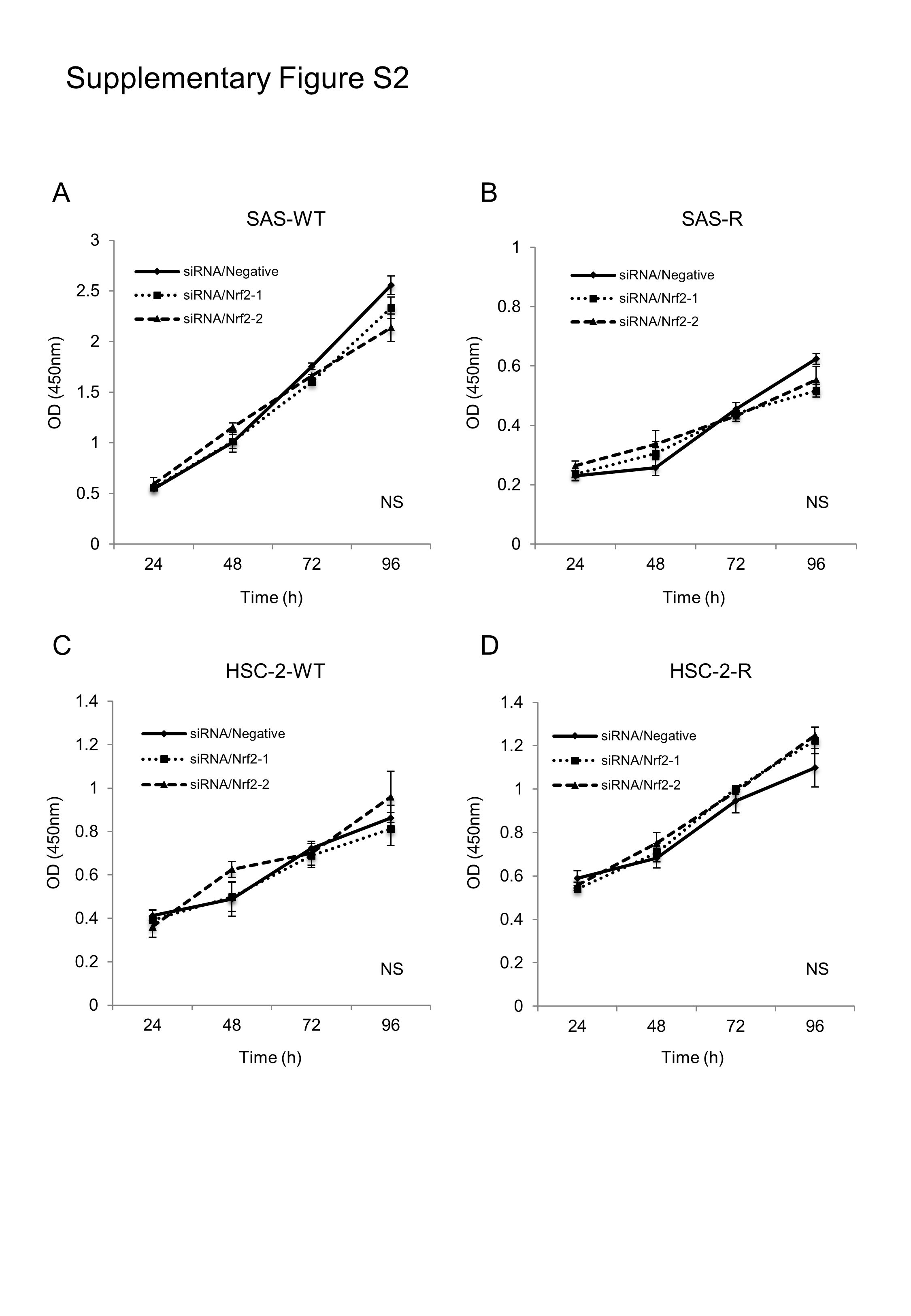

Supplement: Supplementary file 2 — Supplementary Figure S2 [file 41374_2022_776_MOESM2_ESM.tif]

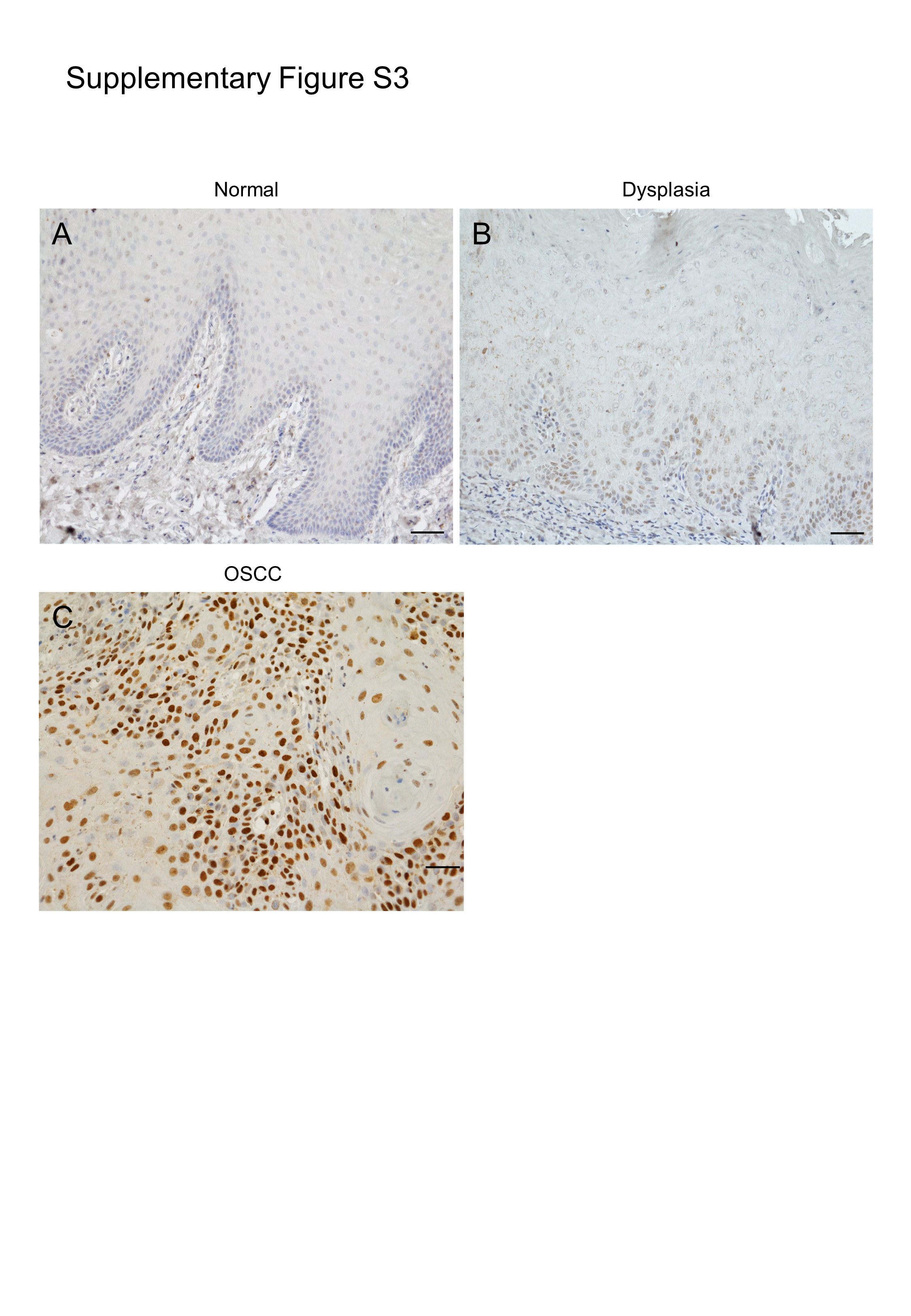

Supplement: Supplementary file 3 — Supplementary Figure S3 [file 41374_2022_776_MOESM3_ESM.tif]
