# Supplementary material for: The antioxidative stress regulator Nrf2 potentiates radioresistance of oral squamous cell carcinoma accompanied with metabolic modulation
Source: Lab Invest. 2022 Apr 12;102(8):896–907. doi: 10.1038/s41374-022-00776-w (PMC9309095; doi:10.1038/s41374-022-00776-w)
Supplement: Supplementary file 4 — Supplemental Figure Legends [file 41374_2022_776_MOESM4_ESM.docx]

**Supplementary information**

**Supplementary Figure S1. The protein expression of Nrf2 in HSC-3, Ca9-22, OSC-20 and HOC-313 cells under normal conditions.**

Whole-cell protein was prepared, and the expression of Nrf2 was examined via Western blotting.

**Supplementary Figure S2. Transfection with small interfering RNA (siRNA) against Nrf2 has no effect on cell proliferation.**

(A) SAS-WT cells. (B) SAS-R cells. (C) HSC-2-WT cells. (D) HSC-2-R cells. The degree of cell proliferation was monitored for 4 days using Cell Counting Kit-8 after siRNA transfection. The results are presented as the mean ± SD of three independent experiments. **P* < 0.05 and ***P* < 0.01.

**Supplementary Figure S3. Immunohistochemical staining of phosphorylated Nrf2 in adjacent normal, dysplastic, and oral cancer sections.**

(A) Normal oral tissue. (B) Oral dysplasia. (C) Oral squamous cell carcinoma. Original magnification, ×200; bar, 50 µm.
